# School performance trajectories and young adult offending: Findings from a national administrative data linkage, United Kingdom

**DOI:** 10.23889/ijpds.v8i2.2199

**Published:** 2023-09-14

**Authors:** Alice Wickersham, Rosie Cornish, Stephen Scott, Johnny Downs

**Affiliations:** 1 King's College London, London, United Kingdom; 2 University of Bristol, Bristol, United Kingdom

## Objectives

Criminal offending and re-offending comes at a significant social and economic cost. Offending prevention therefore presents a high priority policy area. Low educational attainment is a known risk factor for offending, but little is understood about how changes in school performance over time might be associated with offending.

## Method

We investigated this in a large sample of n~4.3 million pupils using an administrative data linkage between two routinely-collected national datasets: the National Pupil Database (NPD) and the Police National Computer (PNC). First, we conducted growth mixture modelling using NPD data over three statutory testing years (School Years 2, 6 and 11). We then investigated the association between membership of these trajectories and subsequent conviction or caution for any criminal offence between Year 11 and age 21.

## Results

We derived five school performance trajectories: (1) Average Consistent (n=3,497,167, 81.0%), (2) Average Increasing (n=66,383, 1.5%), (3) Average Declining (n=373,117, 8.6%), (4) Low Increasing (n=98,805, 2.3%), and (5) Low Consistent (n=281,964, 6.5%). The Average Declining group had the highest proportion of individuals who went on to be convicted or cautioned for any first offence up to age 21 (9.8%), followed by the Low Consistent (8.5%), Low Increasing (5.6%), Average Consistent (4.2%) and Average Increasing (1.5%) groups. Furthermore, as the number of offending days between Year 11 and age 21 increased (indicating repeat offending), the likelihood of having been in the Average Declining or Low Consistent groups also increased. We will also present findings from multilevel models accounting for school clustering, different offence types, and interactions.

## Conclusion

Tentatively, findings suggest that changes in school performance could help to identify pupils who are struggling and at increased risk of criminal justice involvement, and therefore might be in need of additional support.

